# iNOS is a key mediator of anti-PD-1 melanoma therapy response

**DOI:** 10.3389/fimmu.2026.1837275

**Published:** 2026-06-22

**Authors:** Quang Tam Nguyen, Youngchul Kim, Thi Hong Nga Le, Saurabh Garg, Vishanna Balkaran, Trisha Bhathivi, Alejandra Chamizo, Christopher W. Dukes, Kimberly Ward, Andrew S. Brohl, Lilit Karapetyan, Nikhil I. Khushalani, Zeynep Eroglu, Ahmad A. Tarhini, James J. Mulé, Joseph Markowitz

**Affiliations:** 1 Department of Cutaneous Oncology, H. Lee Moffitt Cancer Center and Research Institute, Tampa, FL, United States; 2 Department of Immunology, H. Lee Moffitt Cancer Center and Research Institute, Tampa, FL, United States; 3 The Immuno-Oncology Program, H. Lee Moffitt Cancer Center and Research Institute, Tampa, FL, United States; 4 Department of Biostatistics and Bioinformatics, H. Lee Moffitt Cancer Center and Research Institute, Tampa, FL, United States; 5 Department of Oncologic Sciences, University of South Florida Morsani School of Medicine, Tampa, FL, United States

**Keywords:** anti-PD-1, immunotherapy, iNOS, melanoma, nitric oxide

## Abstract

**Background:**

Inducible nitric oxide synthase (iNOS) and its product nitric oxide (NO) were historically linked to poor melanoma outcomes, yet recent evidence shows NO supports anti-tumor immunity. This study examines how iNOS shapes anti–PD-1 efficacy, particularly through interferon signaling.

**Methods:**

B16-D5 melanoma tumors were implanted in wild-type (WT) and iNOS knockout (KO) mice to compare tumor growth and response to anti–PD-1 therapy. Flow cytometry, apoptosis assays, and RNA sequencing assessed NO production, PD-L1 expression, and interferon-related gene activation. *In vitro*, melanoma cells were treated with NO donors (DETA NONOate, SNAP) to assess proliferation and apoptosis. Peripheral blood mononuclear cells from 27 melanoma patients receiving anti–PD-1 therapy were analyzed with multiparameter flow cytometry to correlate NO-associated immune subsets with progression-free survival (PFS).

**Results:**

Tumors grew significantly faster in iNOS KO mice, and anti–PD-1 therapy had no effect, demonstrating that iNOS-derived NO contributes to treatment efficacy. NO donors inhibited melanoma proliferation and induced apoptosis *in vitro*. Transcriptomic analysis showed anti–PD-1 upregulated interferon pathway genes (STAT1, IRF1, IFNB1) in WT but not iNOS KO mice. In patients, a NO-producing dendritic-cell subset (DAF-FM^+^CD11c^+^) was associated with improved PFS (hazard ratio 0.453; 95% CI = 0.270-0.992; p=0.048), indicating a NO-dependent enhancement of interferon-driven immune activity.

**Conclusion:**

iNOS-derived NO is necessary for effective anti–PD-1 immunotherapy in melanoma, promoting interferon signaling and immune activation. Loss of iNOS impairs tumor control and immune responsiveness, supporting NO as a potential biomarker and therapeutic adjunct to be explored while challenging assumptions about its deleterious role in melanoma.

## Introduction

The presence of cytokine-inducible nitric oxide (NO) biosynthesis in humans was conclusively established during interleukin 2 melanoma therapy (1). There is growing evidence that NO has a dichotomous effect on melanoma depending on its concentration and source, being melanoma itself or infiltrating immune cells (i.e., T cells), with different putative effects based on immune-based treatments (2–6). Upwards of 60% of patients can respond to combination checkpoint blockade in melanoma, but this is only 40% with single-agent anti-PD-1, albeit with significantly less toxicity (7, 8). Understanding the mechanisms of response and resistance to anti-PD-1 therapy is crucial for enhancing efficacy and outcomes in melanoma patients.

Nitric oxide has long been linked to melanoma, with iNOS levels and biochemistry reviewed elsewhere (5). Subsequently, it was found that anti-PD-1 functions partially by stimulating a type I interferon effect (9). PD-L1 expression is known to be enhanced with increased interferon (IFN) signaling in the setting of checkpoint blockade (9). Our prior studies showed that elevated STAT1 nitration, a key player in type I immune responses, is associated with improved relapse-free survival in ipilimumab-treated patients (3). Additionally, our analysis of proteomic profiles from FFPE samples collected before anti-PD-1 therapy suggested that iNOS plays a central role in distinguishing good responders from poor responders (4). This study investigated the role of nitric oxide (NO) in melanoma models treated with anti-PD-1 and revealed, for the first time, that anti-PD-1 therapy is ineffective without NO, which is linked to an IFN response.

## Methods

### Culture medium and tumor cell line

Complete medium (CM) consisted of RPMI 1640 supplemented with 10% heat-inactivated FCS, 0.1 mM nonessential amino acids, 1 μM sodium pyruvate, 2 mM fresh l-glutamine, 100 μg/ml streptomycin, 100 U/ml penicillin, 50 μg/ml gentamycin, 0.5 μg/ml fungizone, and 0.05 mM 2-mercaptoethanol (Thermofisher, Waltham, MA). A poorly immunogenic, highly metastatic subclone of the murine melanoma cell line B16-D5, herein denoted B16, has been previously characterized (10).

### Measurement of PD-L1 expression on B16 melanoma cells

B16-D5 cells (2 × 10^4^) were seeded in 24-well plates and incubated for 24 hours. Cells were then treated with varying concentrations of DETA-NONOate, a nitric oxide donor (#AC328650250, ThermoFisher, Waltham, MA) for 24 hours, followed by trypsinizing and staining with BV421-conjugated anti-mouse PD-L1 (CD274, #564716, BD Biosciences, Franklin Lakes, NJ) or isotype control (#562965, BD Biosciences). Flow cytometry was performed using a BD™ LSR II system.

### CFSE cell proliferation assay and DAF-FM measurement by flow cytometry

B16-D5 cells were stained with 5 μM CFSE (#423801, BioLegend, San Diego, CA) for 20 min at 37 °C per manufacturer’s instructions. Excess dye was removed by washing with five volumes of CM. Then, 2 × 10^4^ B16-D5 cells were seeded per well in a 24-well plate. The next day, varying concentrations of DETA NONOate or SNAP were added. After 48 hours, cells were trypsinized, washed, and stained with Zombie NIR fixable viability dye (#423106, BioLegend, San Diego, CA). Flow cytometry was performed using the BD™ LSR II system. Since CFSE halves with each division, live single cells (Zombie NIR–negative) were gated, and CFSE mean fluorescence intensity was used to assess proliferation.

DAF-FM (4-amino-5-methylamino-2’,7’-difluorofluorescein) was used as a fluorescent probe for nitric oxide detection. To quantify nitric oxide levels within tumor microenvironment cell populations, C57BL/6J mice were inoculated subcutaneously with 1 × 10^5^ B16 melanoma cells and treated as indicated. Tumors were subsequently excised, and single−cell suspensions were prepared. Single-cell tumor suspensions were incubated with 150 nM DAF-FM (#D23844, Invitrogen, Waltham, MA) as previously described (2), then stained with CD45-PE-Cy-5 (#394612, BD Biosciences, Franklin Lakes, NJ) and analyzed using the BD™ LSR II Flow Cytometer System.

### Apoptosis assay

B16-D5 melanoma cells (2 × 10^4^) were seeded in 24-well plates and allowed to adhere for 24 hours. Media were replaced, and cells treated with varying concentrations of DETA NONOate or SNAP. After 48 hours, the cells were trypsinized and apoptosis was assessed via flow cytometry using the FITC Annexin V Apoptosis Detection Kit I (#556547, BD Biosciences, Franklin Lakes, NJ) per manufacturer’s instructions. Cells were washed with PBS, resuspended in 1X binding buffer, stained with 5 µl Annexin V-FITC and 5 µl PI, incubated for 15 minutes at room temperature in the dark and measured by flow cytometry.

### Murine experiments

C57BL/6J and iNOS KO mice were obtained from Jackson Laboratories (Bar Harbor, ME) at 4 weeks of age and housed for 2 weeks under institutional IACUC approval. B16-D5 cells were injected subcutaneously into the flank of mice 8 days before treatment (day -8) to allow tumors to establish. Day 0 marks the initiation of therapy. Mice were randomly assigned to three groups: control (no treatment), IgG-treated (#BE0089, BioXCell, Lebanon, NH), and anti-PD-1-treated (#BE0146, BioXCell, Lebanon, NH). Treatments (250 µg/200 µL PBS) were administered on Days 0 and 3. Tumor sizes were measured with electronic calipers, and tissues were collected at various post-treatment time points, snap-frozen, and stored at −80 °C.

### RNASeq measurement and processing of data

Fresh tumor tissue was processed for total RNA extraction using the RNeasy Blood & Tissue Kit (Qiagen, Germantown, MD) per the manufacturer’s instructions. RNA was quantified with the Qubit Fluorometer (ThermoFisher Scientific, Waltham, MA) and assessed for quality using the Agilent TapeStation 4200 (Agilent Technologies, Santa Clara, CA). Samples were prepared for RNA sequencing with the NuGEN Universal Mouse RNA-Seq Library Preparation Kit with NuQuant (Tecan Genomics, Redwood City, CA). Using 100 ng of RNA, cDNA and strand-specific libraries were generated following the manufacturer’s protocol. Quality control included TapeStation size assessment and quantification via the Kapa Library Quantification Kit (Roche, Wilmington, MA). Final libraries were normalized, denatured, and sequenced on the Illumina NovaSeq 6000 using 200-cycle reagent kits, yielding ~60 million 100-base read pairs per sample (Illumina, Inc., San Diego, CA).

### Flow cytometry of PBMCs from melanoma patients

Peripheral blood samples were collected from 27 patients receiving pembrolizumab or nivolumab for metastatic melanoma under protocol MCC 19634 approved by the Advarra^®^ Institutional Review Board. A flow cytometry panel was used to analyze PBMCs prior to anti-PD-1 treatment, including markers for DAF-FM (nitric oxide), CD3, CD4, CD8, CD25, CD127, CD56, CD19, and CD11c. Controls included compensation beads for matrix calibration, fluorescence minus one (FMO) controls for gating, isotype controls to account for patient variability, and a live/dead marker. Cell phenotypes were determined using MPATR, a multi-parameter phenotyping analysis tool developed by our group (2).

### Bioinformatics analysis

For RNASeq data, read adapters were detected using BBMerge (v37.02) (11) and subsequently removed with cutadapt (v1.8.1) (12). Processed raw reads were then aligned to mouse genome mm10 using STAR (v2.7.7a) (13). Gene-level expression was estimated as read count using RSEM (v1.3.0) (14) and GENCODE gene model vM21. Gene expression data were then normalized and differential expression between experimental groups were evaluated using DESeq2 (15). A false-discovery rate was controlled at 0.05 using a multiplicity test. Qiagen Ingenuity Pathway Analysis software was used to analyze the data from the RNASeq analysis, employing standard techniques (16, 17). To examine the relationship between NO levels in immune cell subtypes and progression-free survival (PFS), we employed Cox proportional hazards regression (CPH) and partial least squares regression (PLS) models of the nodes in the clustering trees as a function of PFS risk.

### Statistical analysis

Statistical analysis of tumor growth curves was performed using a CGGC permutation test. CFSE, apoptosis, PD-L1, and NO levels were summarized using mean and standard deviation. Group differences were assessed using two-sample t-tests or Wilcoxon rank-sum tests, selected based on distribution normality. For comparisons among three or more groups, one-way ANOVA followed by Dunnett’s *post-hoc* test was used. Paired t-tests or Wilcoxon signed-rank tests evaluated differences between time points.

## Results

### Reduced nitric oxide production promotes melanoma tumor growth *in vivo*

**Figure 1:**
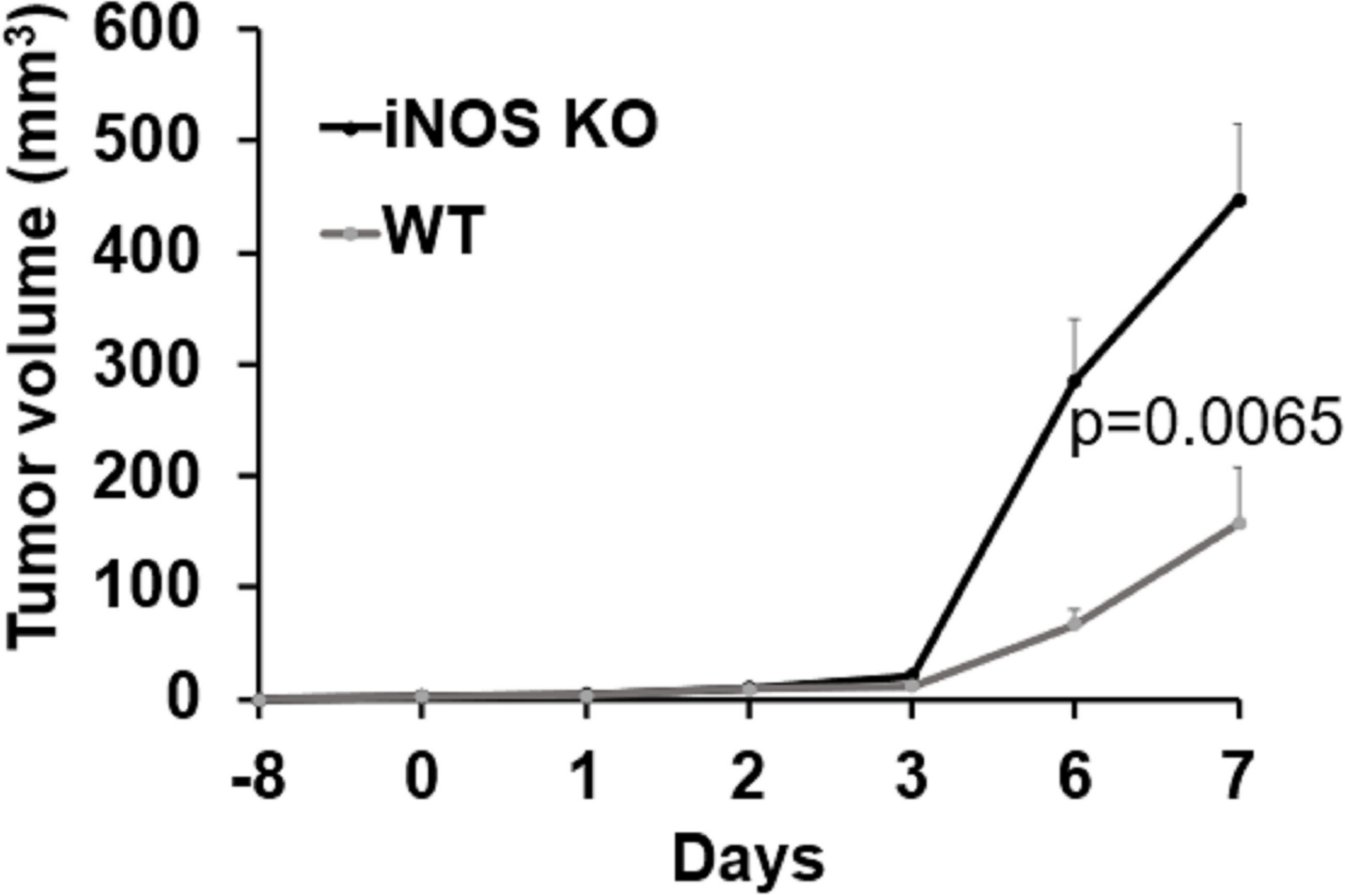
Accelerated melanoma growth in iNOS KO mice. C57BL/6J WT or iNOS KO mice were subcutaneously injected into the flank with 1x10^5^ B16-D5 melanoma cells on day -8. Tumor sizes were measured daily (n = 6 mice per group). Statistical analysis of tumor growth curves was performed using a CGGC permutation test (p < 0.01), representative of 2 experiments performed.

We first investigated NO involvement in regulating tumor growth. As shown in **Figure 1**, tumor growth accelerated more rapidly in iNOS KO mice compared to the wild-type WT control group (p = 0.0065), suggesting that NO production is a negative regulator of melanoma growth *in vivo.*

### Nitric oxide inhibits proliferation and induces apoptosis of melanoma cells *in vitro*

**Figure 2:**
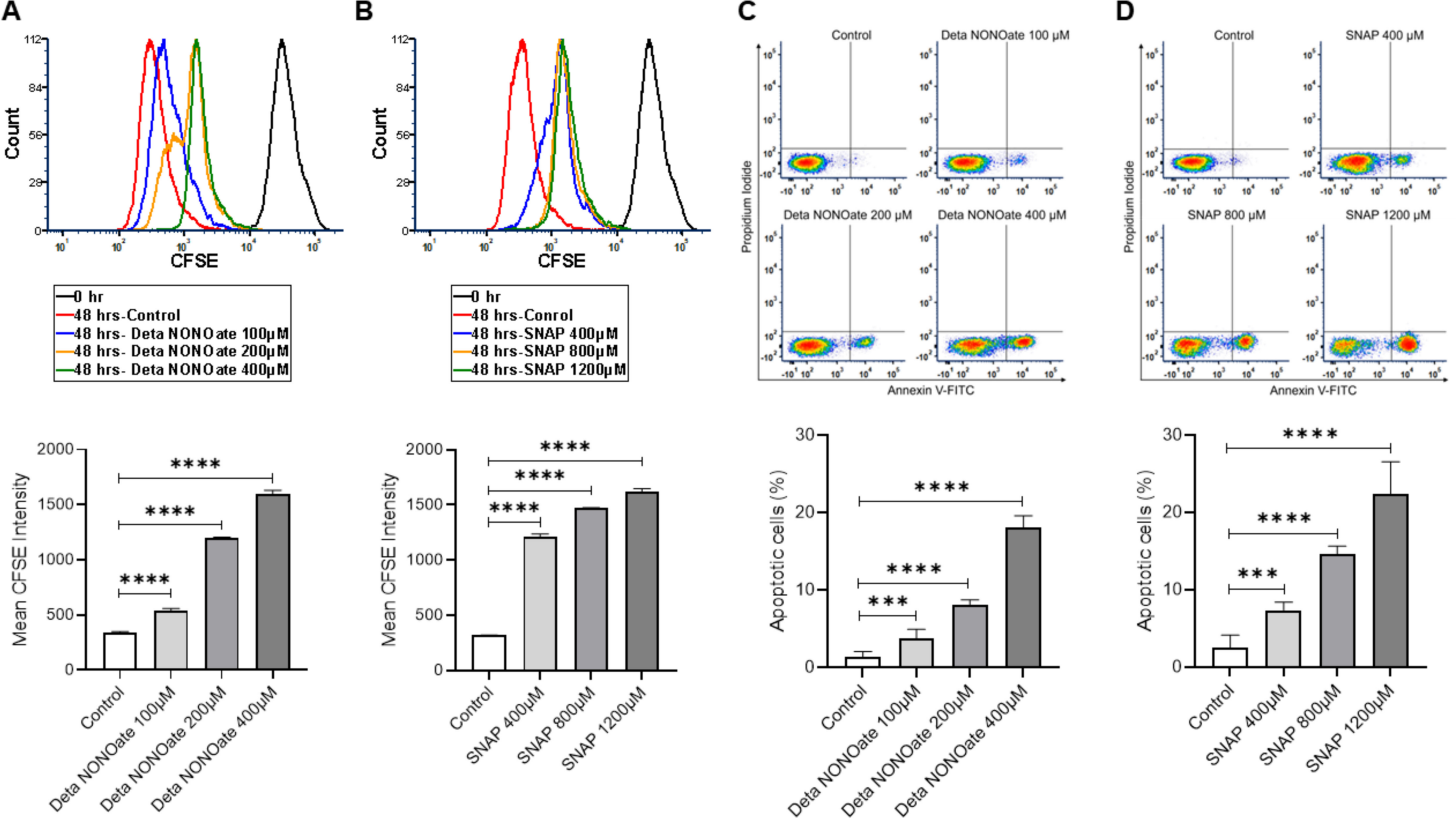
Treatment with NO donors inhibits growth and induce apoptosis of B16 melanoma cells *in vitro*. **(A, B)** B16-D5 melanoma cells were incubated with 5 µM CFSE. The CFSE-labeled cells were washed, incubated for 24 hours, and then treated with various concentrations of either DETA NONOate **(A)** or SNAP **(B)** for 48 hours. Cell division was monitored by flow cytometry. Representative histograms (top panels) and mean CFSE intensity (bottom panels) are shown. **(C, D)** B16 cells were treated with DETA NONOate **(C)** or SNAP **(D)** for 48 hours, followed by staining with Annexin V-FITC and propidium iodide (PI). Representative flow cytometry dot plots (top panels) and quantification of apoptotic cells in the total cell population (bottom panels) are shown. Data are from 3 independent experiments. Ordinary one-way ANOVA with Dunnett’s multiple comparisons test was used to analyze the data. ***p < 0.001, ****p < 0.0001. Deta NONOate, Diethylamine NONOate; SNAP, S-Nitroso-N-acetylpenicillamine.

To assess nitric oxide’s effect on melanoma proliferation, two NO donors were tested: DETA NONOate which releases NO spontaneously, and SNAP which generates NO via a thiol-dependent mechanism (18). CFSE-labeled B16 melanoma cells were treated with varying donor concentrations for 48 hours. Flow cytometry showed DETA NONOate significantly inhibited proliferation in a dose-dependent manner (**Figure 2A**, p < 0.0001). SNAP markedly reduced melanoma cell proliferation at a concentration of 400 µM (p<0.0001), with the inhibitory effect reaching a plateau between 800 and 1200 µM (**Figure 2B**).

A previous study showed that NO donors induce apoptosis in human melanocytes (19). To assess whether reduced melanoma proliferation involves apoptosis, B16 melanoma cells were treated with DETA NONOate or SNAP (**Figures 2C, D**), followed by Annexin V/PI staining. Both NO donors significantly increased apoptosis compared to untreated controls. DETA NONOate (400 µM) caused a ~13-fold rise in apoptotic cells (18.1% vs. 1.3%), while SNAP showed a dose-dependent effect, with 7.3% apoptosis at 400 µM and 22.4% at 1200 µM (**Figures 2C, D**). These results indicate that NO donors directly induce apoptosis in B16 melanoma cells.

Our findings show that NO donors suppress B16 melanoma cell growth by inducing apoptosis. Reduced viability and increased Annexin V^+^ apoptotic populations in a dose-dependent manner indicate that NO anti-proliferative effects are primarily apoptotic rather than cytostatic.

In the context of immune checkpoint blockade, NO–induced apoptosis may offer dual benefits: reducing tumor burden and increasing immunogenic antigen availability. This could potentiate PD-1 blockade by strengthening cytotoxic T cell responses. Thus, NO donors may serve as effective adjuncts to immunotherapy, combining direct tumoricidal action with immune enhancement.

### Anti-tumor effect of anti-PD-1 treatment is NO dependent

To determine whether anti-PD-1 treatment modulates NO production, we measured NO levels in B16-D5 tumor-bearing WT mice treated with anti-PD-1 antibodies. Administration of anti-PD-1 led to a marked increase in NO production in both CD45^+^ immune cells and CD45^-^ non-immune cells within the tumor, as indicated by elevated DAF-FM fluorescence levels at 6 hours post-treatment (**Supplementary Figure 1**). It should be acknowledged that DAF-FM binds to NO and NO metabolites. It is possible that this transient elevation is partly contributed by NO metabolites. Therefore, iNOS KO mice were used throughout the study to eliminate the effects of NO produced by iNOS in the animal model.

**Figure 3:**
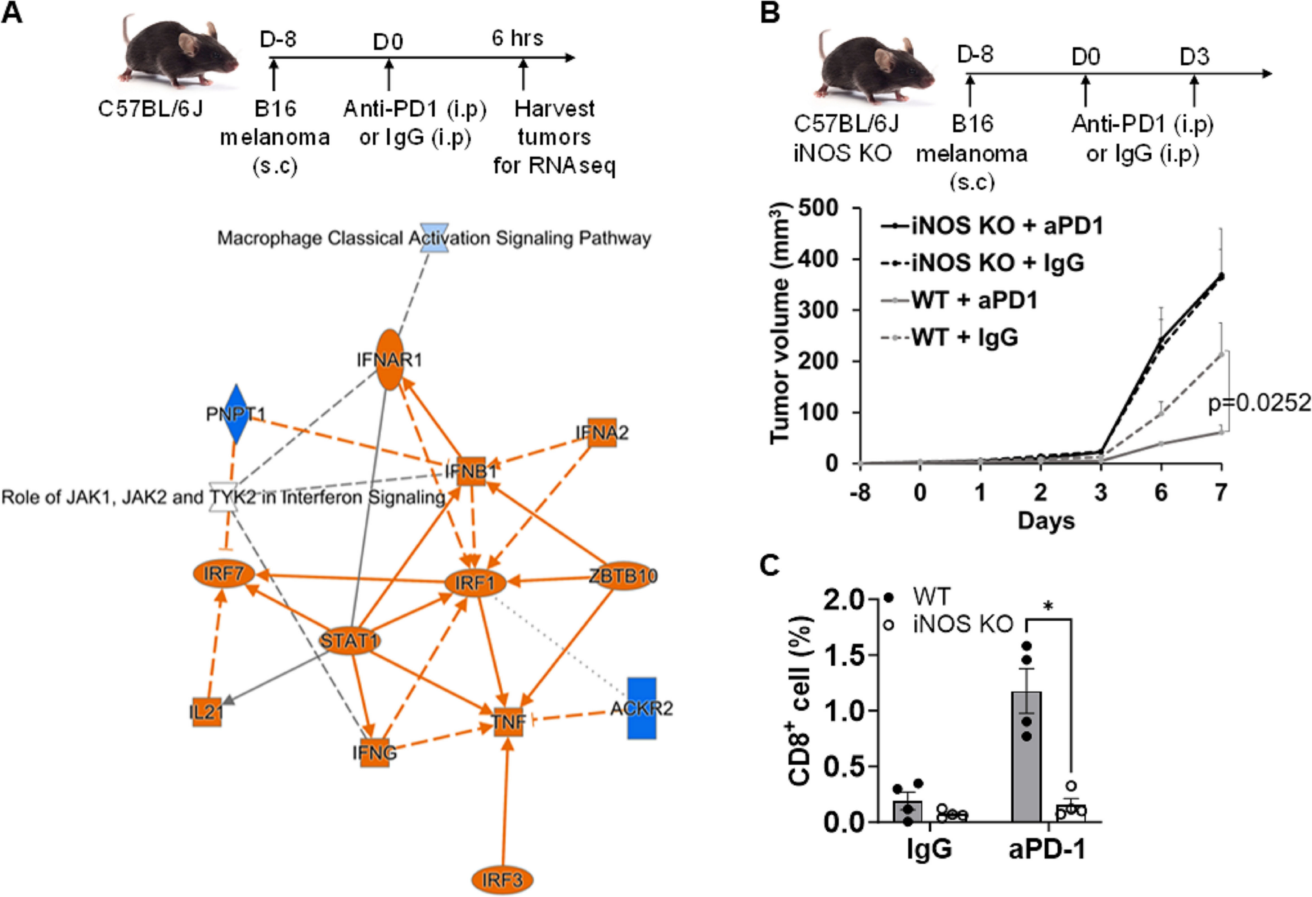
Anti-tumor effect of anti-PD-1 antibody treatment is abolished in iNOS KO mice. C57BL/6J WT or iNOS KO mice were subcutaneously injected with 1x10^5^ B16-D5 melanoma cells 8 days prior to administration of either anti-PD-1 or IgG control antibodies (250 µg/mouse). **(A)** Timeline of the experiment (top panel) and transcriptomics of cells isolated from tumor masses at 6 hours post-treatment of antibodies (bottom panel), followed by pathway analysis (RNASeq performed in triplicate using individual tumors from three animals per condition). **(B)** Experimental timeline diagram for animal studies (top panel) and tumor growth curve (bottom panel). Tumor induction was performed as in **(A)**. Administration of anti-PD-1 or IgG control antibodies (250 µg/mouse) was conducted on Day 0 and day 3 (top panel). Tumor sizes were measured daily (bottom panel) (n = 7 mice per group; experiment performed in duplicate). Statistical analysis of tumor growth curves was performed using a CGGC permutation test. **(C)** Tumors from experiment in **(B)** were stained for CD8 cells and quantified using QuPath. Multiple t-test with Welch correction was used to analyze data. *p<0.05.

To further explore molecular responses to anti-PD-1 therapy, we performed pathway analysis on transcriptomic data from B16-D5 tumors in WT mice 6 hours post-treatment, comparing them to IgG and untreated controls. Informatics gene pathway and network analysis revealed a significant upregulation of key transcription factors (STAT1, IRF1, IFNG, and IFNB1), central to type I and II interferon signaling in tumors from WT mice 6 hours post–anti-PD-1 treatment (**Figures 3A**, orange), indicating pathway activation in response to PD-1 blockade. In this respect, anti-PD-1 therapy is known to increase type II interferon responses (9). Together, these data revealed that anti-PD-1 treatment induced NO production in the tumor microenvironment and enhanced early interferon signaling.

To confirm NO’s role in anti-PD-1 efficacy, WT and iNOS KO mice were treated with control IgG or anti-PD-1 antibodies. The anti-tumor effect of anti-PD-1 therapy was eliminated in the iNOS KO mice, as indicated by overlapping tumor growth curves for anti-PD-1 and IgG treated iNOS KO mice (**Figure 3B**). Moreover, anti-PD-1–induced CD8 T cell increase in WT mice was absent in iNOS KO mice (**Figure 3C**). Together, this data demonstrated that the presence of iNOS and the resulting nitric oxide production are necessary for the efficacy of anti-PD-1 therapy.

### NO contributes to an optimal interferon-dependent response to anti-PD-1 therapy

**Figure 4:**
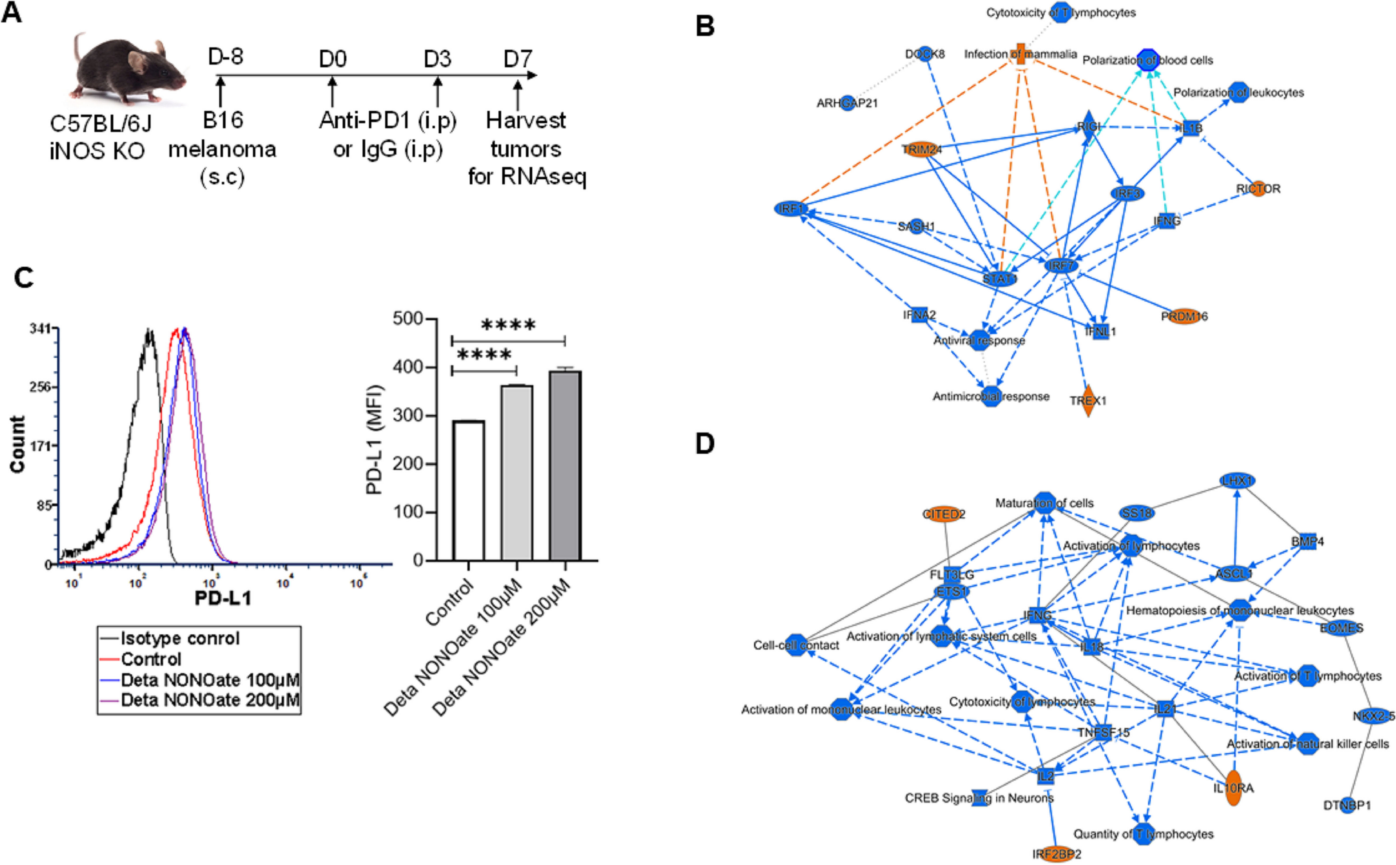
Anti-PD-1 antibody treatment in iNOS KO mice results in reduced interferon responses. C57BL/6J WT or iNOS KO mice were subcutaneously injected with 1x10^5^ B16-D5 melanoma cells 8 days prior to administration of either anti-PD-1 or IgG control antibodies (250 µg/mouse) (n = 7 mice per group, experiment performed in duplicate). **(A)** Timeline of the experiment. **(B)** Whole transcriptomic analysis of tumors ± anti-PD-1 in iNOS KO mice at 7 days post-treatment of antibodies, followed by pathway analysis. Tumors were processed individually, n = 3 mice per group at study endpoint. **(C)** Flow cytometric analysis of cell surface PD-L1 expression in B16 melanoma cells following treatment for 24 hrs with varying concentrations of DETA-NONOate. Representative histograms (left panel) and mean fluorescence intensity (MFI) of PD-L1 expression (right panel) are shown. Data were collected from 2 independent experiments. Ordinary one-way ANOVA with Dunnett’s multiple comparisons test was used to analyze the data. ****p < 0.0001. **(D)** Whole transcriptome analysis was performed on tumor masses from anti-PD-1-treated WT and anti-PD-1-treated iNOS knockout mice 7 days after antibody treatment, followed by pathway analysis. Tumors were processed individually, n = 3 mice per group at study endpoint.

To investigate signaling pathways affected by iNOS deficiency during anti-PD-1 therapy, tumors were injected subcutaneously into the flank, and treatment started on day 0 (**Figure 4A**). Transcriptomic analysis revealed significantly reduced activation of interferon-related genes in anti-PD-1–treated iNOS KO mice compared to WT (**Figure 4B**). Gene pathway and network analysis showed that iNOS deficiency suppressed multiple interferon-regulated pathways, with key transcription factors (STAT1, IRF1, IRF3, and IRF7) downregulated (blue). This effect was expected as STAT1 binds to the iNOS promotor (20). These findings show that anti–PD-1 therapy triggers a nitric oxide–dependent interferon response in the tumor microenvironment, and that iNOS deficiency impairs both this response and results in lack of therapeutic efficacy.

Recent studies have demonstrated that IFN signaling regulates PD-L1 expression on melanoma cells (21). To examine the link between nitric oxide (NO), IFN signaling, and PD-L1 expression, we tested whether elevated NO levels upregulate PD-L1 as a functional marker of IFN activity. B16-D5 melanoma cells treated with increasing concentrations of Deta NONOate showed significantly enhanced PD-L1 expression at 200 μM (**Figure 4C**). Extending these findings, transcriptomic analysis of tumors from anti–PD-1–treated WT and iNOS KO mice revealed reduced cytotoxic activity and IFN-γ response in the iNOS-deficient group (**Figure 4D**), reinforcing NO’s essential role in anti–PD-1 efficacy.

Collectively, these findings indicate that NO contributes to an optimal interferon-dependent response to anti-PD-1 therapy. In the absence of iNOS, anti-PD-1 treatment elicits a muted interferon signature and fails to effectively control tumor growth, highlighting a critical role for NO in modulating the efficacy of immune checkpoint blockade.

## Discussion

Early doses of anti–PD-1 therapy are critical for assessing patient response, with some achieving complete remission after just one pembrolizumab treatment (22). However, obtaining these early samples can be challenging in clinical practice. Anti-PD-1 therapy has been shown to increase IFN responses (9). Interferon and STAT1 signaling lead to increased iNOS production (20). Reactive nitrogen species are one mechanism of promoting melanoma cell death (23). Interestingly, while iNOS has historically been associated with poor responses, our published data indicate that elevated levels of protein nitration can lead to improved responses to ipilimumab (3). This dichotomy is supported by literature, as continuous interferon expression contributes to melanoma growth and metastasis (24).

Given the upregulation of IFN pathways, we hypothesized that a NO-producing cell type would be elevated prior to anti-PD-1 therapy. To test this, we analyzed peripheral blood samples from 27 melanoma patients before treatment with pembrolizumab or nivolumab (median age, 74; 59.3% male) using flow cytometry and clustered cells via our MPATR algorithm. PLS regression analyses identified a significant two-component PLS model that distinguished between high- and low-risk patients (p = 0.00068) (**Supplementary Figure 2A**, **Supplementary Table 1**). CPH regression was also performed for each phenotype. Immune cells expressing NO with potential regulatory function were associated with poor response (Node X39 – CD127^low^CD4^+^T cell, HR 1.721(also in top 10 loading components), 95% confidence interval (CI)= 1.082-2.736, p = 0.022; Node X153 - CD127^low^CD8^+^T cell, HR 1.677, 95% CI: 1.023-2.75, p = 0.040; Node X83 - CD56^int^CD11c^+^ NK cell, HR = 1.795, 95% CI = 1.02-3.157, p =0.042) whereas a classical dendritic cell (Node X29 - CD11c+; HR 0.453, 95% CI = 0.207-0.992, p =0.048) was associated with good response (**Supplementary Figure 2B**). Notably, cells expressing NO associated with poor response exhibit markers of potential regulation and have low interferon-producing potential, whereas dendritic cells can mount an interferon-dependent response and differentiation (25), which supports their sensitivity to anti-PD-1 therapy. Our findings underscore that NO, produced via iNOS, is essential for effective anti–PD-1 therapy.

These findings refine our understanding of nitric oxide, suggesting that a robust NO response may contribute to anti-PD-1 therapeutic efficacy in a manner that is highly dependent on cellular and environmental context. To assess systemic relevance, we analyzed peripheral blood mononuclear cells (PBMCs) from melanoma patients, comparing them with observations from the murine model, and found that interferon-dependent cells producing nitric oxide are present in the circulating PBMCs of melanoma patients with increased PFS. Our conclusions were largely based on a commonly used immunotherapy B16 murine model. However, validation in additional tumor models and human systems will be important to substantiate the roles of NO during treatment. We are actively investigating whether this work can uncover a novel therapeutic strategy or predictive biomarker that could transform the way patients are selected for anti–PD–1 therapy and improve clinical outcomes.

## Data Availability

The RNA sequencing datasets generated for this study have been deposited in the NCBI Gene Expression Omnibus (GEO) under accession number GSE334585. The MPATR flow cytometry/data for survival analysis and histology images supporting the findings of this study are publicly available in the Immunology Database and Analysis Portal under study accession SDY3667. Please contact the corresponding author for other manuscript requests.
