# Supplementary figures and images for: iNOS is a key mediator of anti-PD-1 melanoma therapy response
Source: Front Immunol. 2026 Jun 22;17:1837275. doi: 10.3389/fimmu.2026.1837275 (PMC13333588; doi:10.3389/fimmu.2026.1837275)

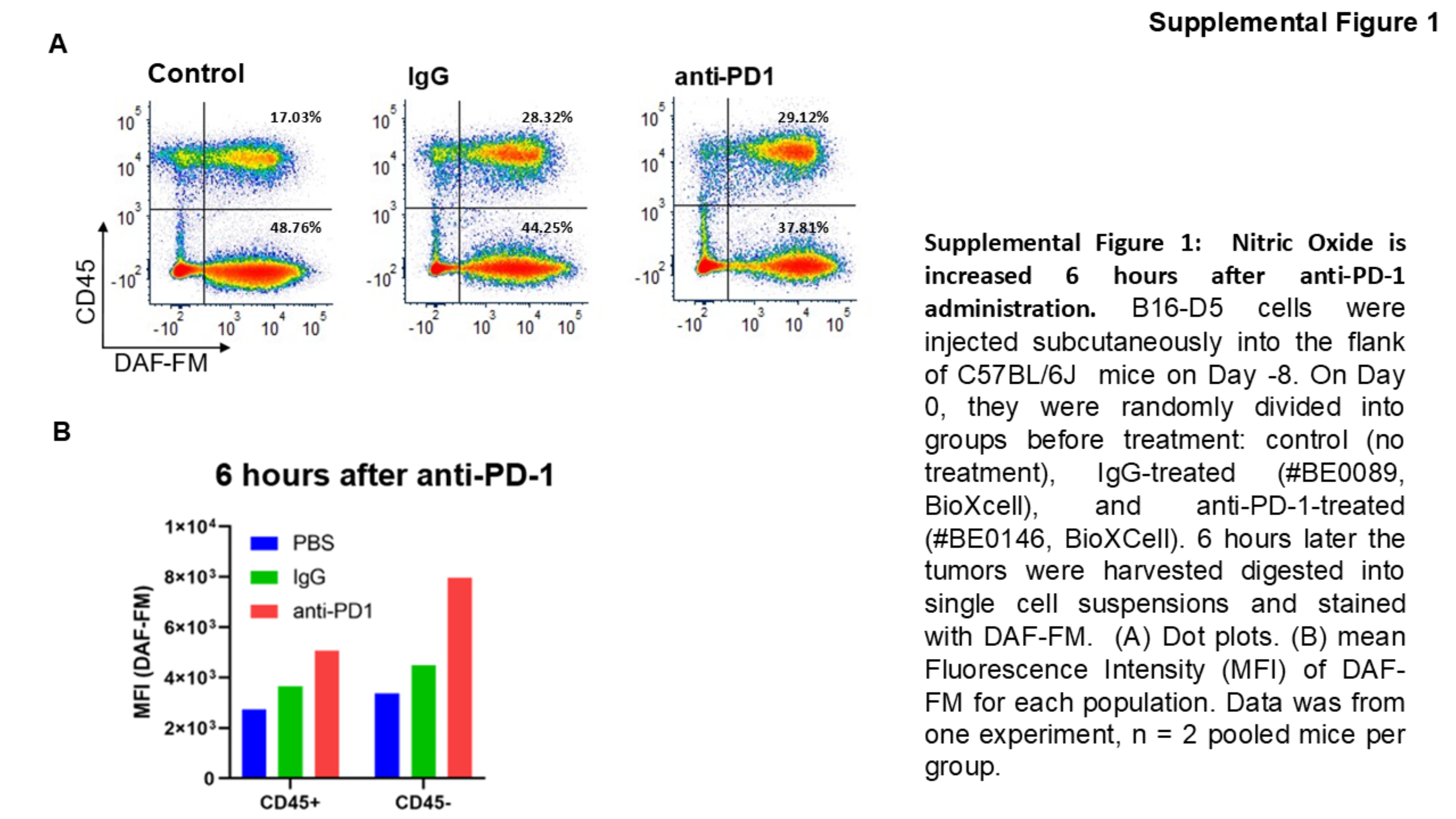

Supplement: Supplementary file 1 [file Image1.tif]

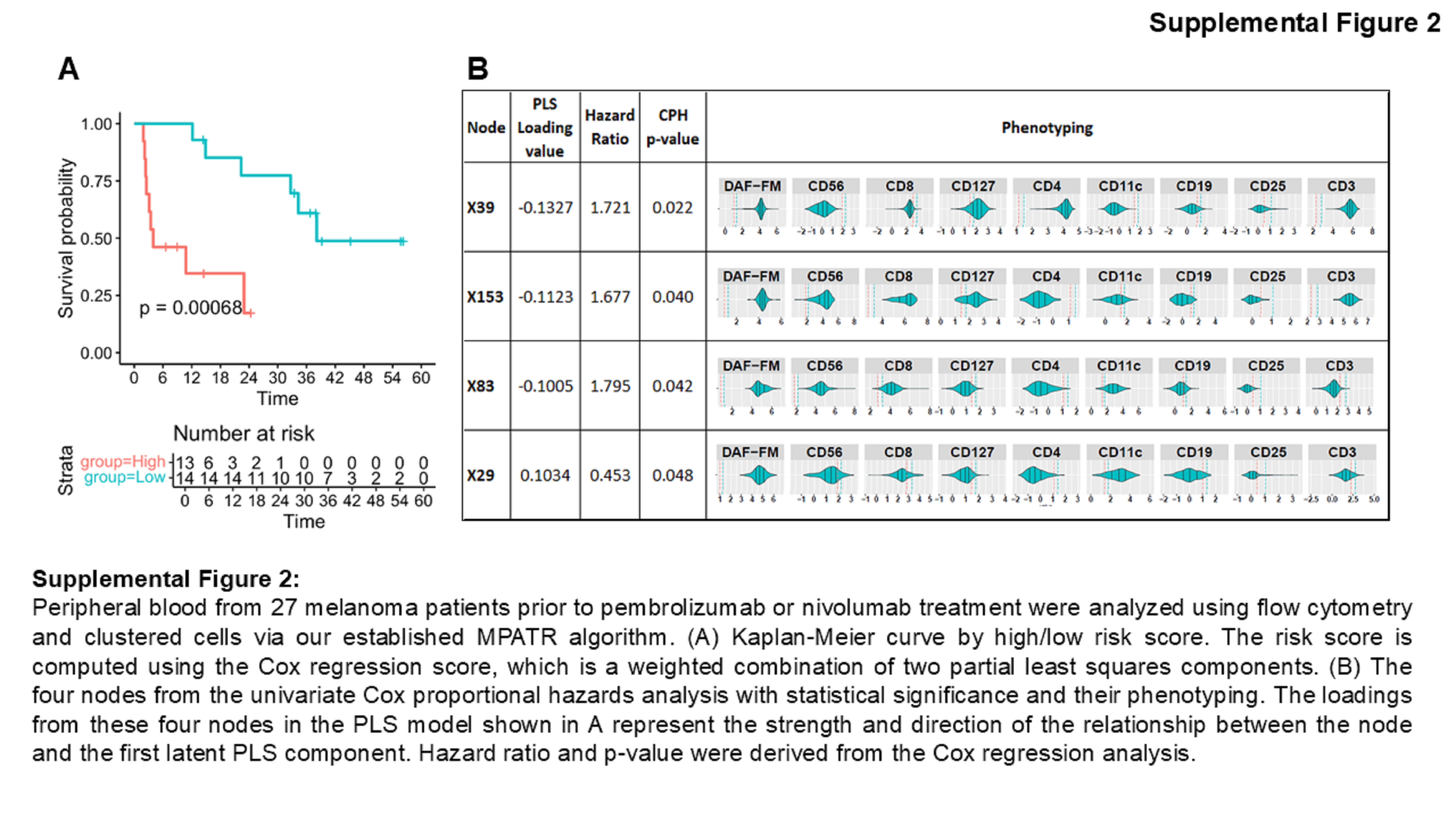

Supplement: Supplementary file 2 [file Image2.tif]

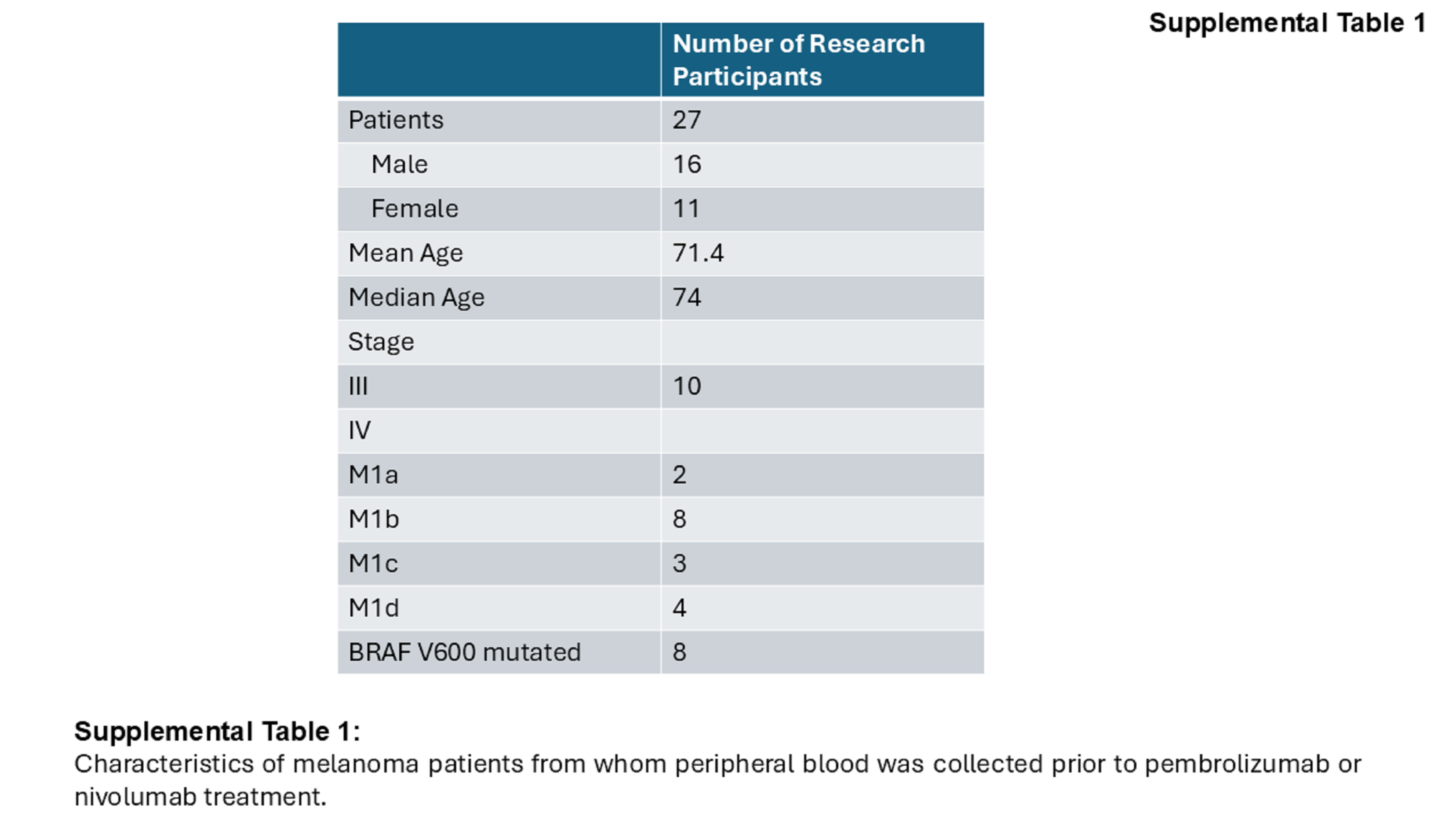

Supplement: Supplementary file 3 [file Image3.tif]
